# Impact of a Commercial Artificial Intelligence–Driven Patient Self-Assessment Solution on Waiting Times at General Internal Medicine Outpatient Departments: Retrospective Study

**DOI:** 10.2196/21056

**Published:** 2020-08-31

**Authors:** Yukinori Harada, Taro Shimizu

**Affiliations:** 1 Department of Diagnostic and Generalist Medicine Dokkyo Medical University Mibu Japan; 2 Department of General Internal Medicine Nagano Chuo Hospital Nagano Japan

**Keywords:** artificial intelligence, automated medical history taking system, eHealth, interrupted time-series analysis, waiting time

## Abstract

**Background:**

Patient waiting time at outpatient departments is directly related to patient satisfaction and quality of care, particularly in patients visiting the general internal medicine outpatient departments for the first time. Moreover, reducing wait time from arrival in the clinic to the initiation of an examination is key to reducing patients’ anxiety. The use of automated medical history–taking systems in general internal medicine outpatient departments is a promising strategy to reduce waiting times. Recently, Ubie Inc in Japan developed AI Monshin, an artificial intelligence–based, automated medical history–taking system for general internal medicine outpatient departments.

**Objective:**

We hypothesized that replacing the use of handwritten self-administered questionnaires with the use of AI Monshin would reduce waiting times in general internal medicine outpatient departments. Therefore, we conducted this study to examine whether the use of AI Monshin reduced patient waiting times.

**Methods:**

We retrospectively analyzed the waiting times of patients visiting the general internal medicine outpatient department at a Japanese community hospital without an appointment from April 2017 to April 2020. AI Monshin was implemented in April 2019. We compared the median waiting time before and after implementation by conducting an interrupted time-series analysis of the median waiting time per month. We also conducted supplementary analyses to explain the main results.

**Results:**

We analyzed 21,615 visits. The median waiting time after AI Monshin implementation (74.4 minutes, IQR 57.1) was not significantly different from that before AI Monshin implementation (74.3 minutes, IQR 63.7) (*P*=.12). In the interrupted time-series analysis, the underlying linear time trend (–0.4 minutes per month; *P*=.06; 95% CI –0.9 to 0.02), level change (40.6 minutes; *P*=.09; 95% CI –5.8 to 87.0), and slope change (–1.1 minutes per month; *P*=.16; 95% CI –2.7 to 0.4) were not statistically significant. In a supplemental analysis of data from 9054 of 21,615 visits (41.9%), the median examination time after AI Monshin implementation (6.0 minutes, IQR 5.2) was slightly but significantly longer than that before AI Monshin implementation (5.7 minutes, IQR 5.0) (*P*=.003).

**Conclusions:**

The implementation of an artificial intelligence–based, automated medical history–taking system did not reduce waiting time for patients visiting the general internal medicine outpatient department without an appointment, and there was a slight increase in the examination time after implementation; however, the system may have enhanced the quality of care by supporting the optimization of staff assignments.

## Introduction

### Background

The waiting time at outpatient departments is directly related to patient satisfaction [1]. Patient distrust regarding medical services increases with longer waiting time, specifically in patients visiting for the first time [1]. Compared to those in other departments, long waiting times in the general internal medicine outpatient departments are, particularly, an issue [2]. Low patient satisfaction may lead to poor patient safety from misunderstandings between patients and medical staff and from medical staff handling patient complaints about waiting time leaving less time for other duties such as medical care. Therefore, reducing waiting time for new patients in general internal medicine outpatient departments may play a vital role in maintaining and improving the quality of medical care. Moreover, reducing the waiting time from arrival in the clinic to the initiation of the medical examination appears to be particularly associated with a reduction of patient anxiety [3].

Clinical documentation is time consuming, taking approximately 34% of physician working time in the outpatient department setting [4]. Moreover, physicians can reduce their clinical evaluation time if summaries of patient histories have already been prepared prior to the examination [5]. Such summaries can be prepared by nurses using self-administered questionnaires provided to patients in the waiting room and completed by hand; this is already widely used in hospitals across Japan. This system, however, has several limitations. First, although a handwritten self-administered questionnaire is a patient-friendly and easy method for medical personnel to collect data, it takes a long time to transfer the detailed information correctly into electronic files. Second, some patients may fill the forms only partially [6], contributing to considerable missing data. Third, the quality of information depends on the skills of the nurse in collecting information. Finally, this system leaves nurses with less time to attend to other professional duties, including engaging in direct patient care [6].

Automated medical history–taking devices appear to be a promising solution for reducing the time spent on transferring handwritten data into digitized form. Automated medical history taking itself has a long history since it was introduced in the late 1960s [7,8]. Until recently, automated medical history taking was used outside of clinics or hospitals and took a long time to complete [9,10], but it has now been implemented in hospital and clinic waiting rooms through computing systems [11,12] and takes only 5 to 10 minutes to complete [11-14]. Its usability and acceptance by patients have been on the rise, and most patients (including older adults) can use automated medical history–taking devices without assistance [11-15]. Automated medical history taking is expected to assist physicians in developing differential diagnoses and to improve on accuracy of diagnoses, though this has not been the case previously [16-18]. Overall, computer-generated patient history recorded by an automated medical history–taking device was reported to be of higher quality, more comprehensive, better organized, and of greater relevance than patient information obtained through traditional methods of medical history taking [19]. Moreover, it was reported to be popular among patients, enabling better communication with physicians, helping to enhance the quality of patient care and making the patients more comfortable in answering sensitive questions [20].

However, there is a paucity of data on the efficacy of automated medical history taking in reducing waiting times. A previous study [21] reported that 45%-60% of physicians believed automated medical history taking could be time saving and efficient because fewer questions need be asked of the patient, less writing is necessary, the automated medical history taking provides a good basis for more detailed questioning, the history is more complete, and patients are forced to think about their problems beforehand [21]. Although not statistically analyzed, some physicians reported average time gained using automated medical history taking was 5 minutes (ranging from none to more than 15 minutes) [21].

### Hypotheses and Study Goal

Recently, an artificial intelligence (AI)–based automated medical history–taking device, AI Monshin, was developed by Ubie Inc in Japan [22]. AI Monshin is not only an automated medical history–taking system but also a clinical decision support system trained to suggest differential diagnoses based on AI machine learning. Based on the positive outcomes of automated medical history–taking devices [21], we hypothesized that replacing the use of handwritten self-administered questionnaires with a new system using AI Monshin would reduce waiting time in a community hospital general internal medicine outpatient department.

## Methods

### Study Design

We conducted a retrospective observational study using data from outpatients who visited the Department of General Internal Medicine at the Nagano Chuo Hospital. The Nagano Chuo Hospital is a medium-sized, secondary community general hospital in Nagano City, Japan and has 332 inpatient beds. The Nagano Chuo Hospital Research Ethics Committee approved the study (serial number: Nagano Chuo Byoin 20-3). The requirement to obtain written informed consent from patients was waived because of the retrospective nature of the study.

### Patient Population

We included patients who had visited the general internal medicine outpatient department in Nagano Chuo Hospital without an appointment between 8 AM and noon on ordinary weekdays (Monday to Friday, excluding hospital holidays) from April 1, 2017 to April 16, 2020. We implemented AI Monshin in the outpatient department on April 17, 2019.

### AI Monshin Tool Presentation

AI Monshin is a software that converts data entered by patients on tablet terminals into technical terms and displays it in the electronic medical record [22]. While in the waiting room, patients enter their age, sex, and symptoms (details can be entered as free text) on a tablet. Consequently, the AI software chooses approximately 20 questions that are tailored to the patient from a pool of 3500 questions. Questions are displayed on the tablet one by one, and patients answer the questions by choosing from the items displayed. The questions are optimized according to previous answers to provide the most relevant list of potential differential diagnoses. It takes approximately 3 minutes to complete the questions [22]. Entered data are summarized and translated into compatible medical text automatically in the patient’s electronic medical record. The top 10 possible differential diagnoses based on history generated by the AI software can be used to assist the physician during patient evaluation.

### Intervention

Different patient flows were applied before and after the introduction of AI Monshin. Before AI Monshin implementation, patients—upon arrival in the clinic—wrote their symptoms, past medical history, family history, social history, and medication history by hand using self-administered questionnaire forms. Upon completion of the form, patients would be interviewed by a nurse, who would check their vital signs, triage the patient, and transfer the patient's information into the electronic medical record system. A doctor would then examine the patient. During and after the examination, the doctor could also edit the patient's medical records using unstructured free text clinical notes.

After AI Monshin implementation, when patients checked in, patients were asked to enter their medical information using the tablet; 5 tablets were introduced. While 3 nurses were engaged in pre-examination interviews prior to the implementation, after the implementation a clerk staff member was hired to assist patients when using the tablets, and one of these nurses was allocated to engaging in nursing work. Clerk staff assisted those who could not use the tablet. After completing the questions on the tablet, nurses would check the vital signs of the patient and triage. Patient data were automatically summarized and translated into compatible medical text in the electronic medical record. The doctor was able to edit the text during and after clinical examination. From the patients' perspective, the difference between before and after AI Monshin implementation were experienced in the waiting and examination rooms. After the implementation of AI Monshin, the patients were only required to fill the electronic form. Patients did not need to wait to be interviewed by a nurse, which usually was the rate-limiting step in outpatient flow prior to AI Monshin implementation. Moreover, patients could see their summary on the monitor in the examination room and could use the displayed information when communicating with doctors. The patient flow after examination was the same before and after AI Monshin implementation.

### Data Collection, Outcomes, and Definitions

We retrospectively collected data, including age, sex, the time of arrival in the hospital, the time of entry into the examination room, and the first registered time of the doctor’s data entry in the patient record for each individual visit. The primary outcome measure was median waiting time per patient. We collected data on waiting time both before and after AI Monshin implementation. The secondary outcome measure was the median waiting time per month. We defined the waiting time as the time between arriving in the hospital and the first recorded time of the doctor’s data entry in the patient record since the time of entry into the examination room was not recorded in all patients.

### Statistical Analyses

We compared the differences in median waiting time before and after AI Monshin implementation using the Wilcoxon rank-sum test. Moreover, we conducted a single-group interrupted time-series analysis [23-25] to evaluate changes in median waiting time per month before and after AI Monshin implementation. We set April 2019 as the start point of implementation. In these analyses, we excluded data with the first recorded time of doctor’s data entry earlier than the time of patient’s arrival in the hospital. Statistical tests were two-tailed, and a *P* value<.05 was considered statistically significant. We conducted all statistical analyses using R (version 3.6.3; The R Foundation for Statistical Computing).

## Results

### Population and Primary Outcome

**Figure 1:**
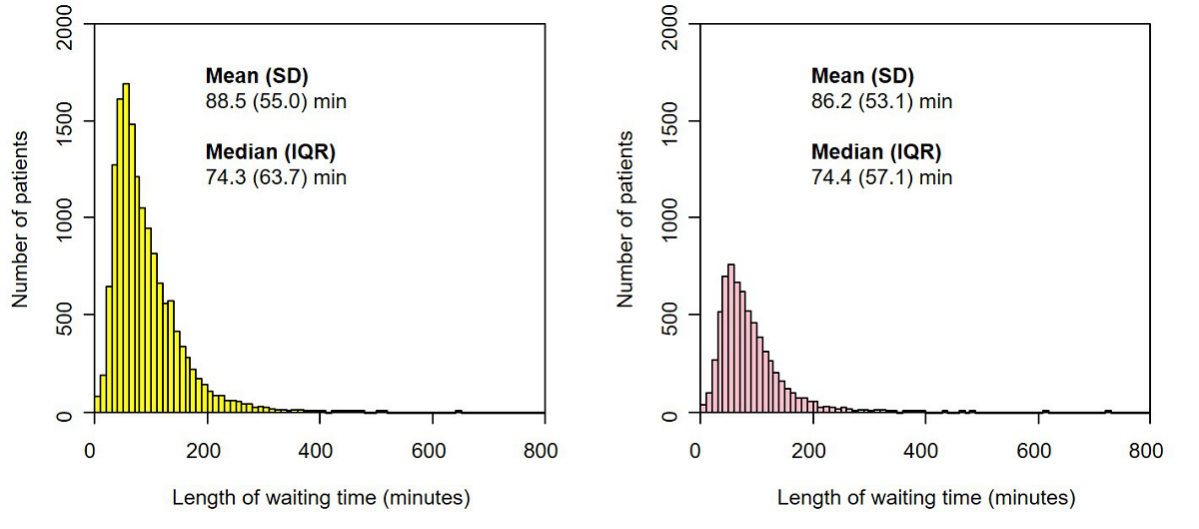
Distribution of waiting time before (left) and after (right) AI Monshin implementation.

From 21,723 eligible patient visits, we excluded 108 (0.5%) because the physicians’ recorded data entry time was earlier than the patient's arrival time (this occurred for patients who did not follow the usual reception process, such as patients who were hospital staff or patients who visited the general internal medicine outpatient department after other departments on the same day). Hence, we included data from 21,615 patients in the study—15,000 patient visits before and 6615 patient visits after the implementation of AI Monshin. Patients who visited preimplementation were significantly older than those who visited postimplementation (age: mean 58.7 versus 56.8 years; *P*<.001). The proportions of men and women and the distribution of arrival times were not significantly different between the two groups (Table 1). Figure 1 shows the distribution of waiting time in the pre (left) and postimplementation (right) groups. Both groups showed the same distribution pattern with an extremely positive skew. The median waiting time was not significantly different between the groups (74.4 minutes versus 74.3 minutes, *P*=.12).

**Table 1:** Characteristics before and after AI Monshin implementation.

Characteristic		Preimplementation (n=15,000)		Postimplementation (n=6615)		*P* value	
Age (years), mean (SD)		58.7 (19.6)		56.8 (20.2)		<.001	
**Gender, n (%)**						.15	
	Men		6801 (45.3)		2930 (44.3)		
	Women		8199 (54.7)		3685 (55.7)		
**Arrival time, n (%)**						.84	
	8 AM-9 AM		4369 (29.1)		1891 (28.6)		
	9 AM-10 AM		4317 (28.8)		1906 (28.8)		
	10 AM-11 AM		3489 (23.3)		1566 (23.7)		
	11 AM-noon		2825 (18.8)		1252 (18.9)		

### Interrupted Time-Series Analysis

**Figure 2:**
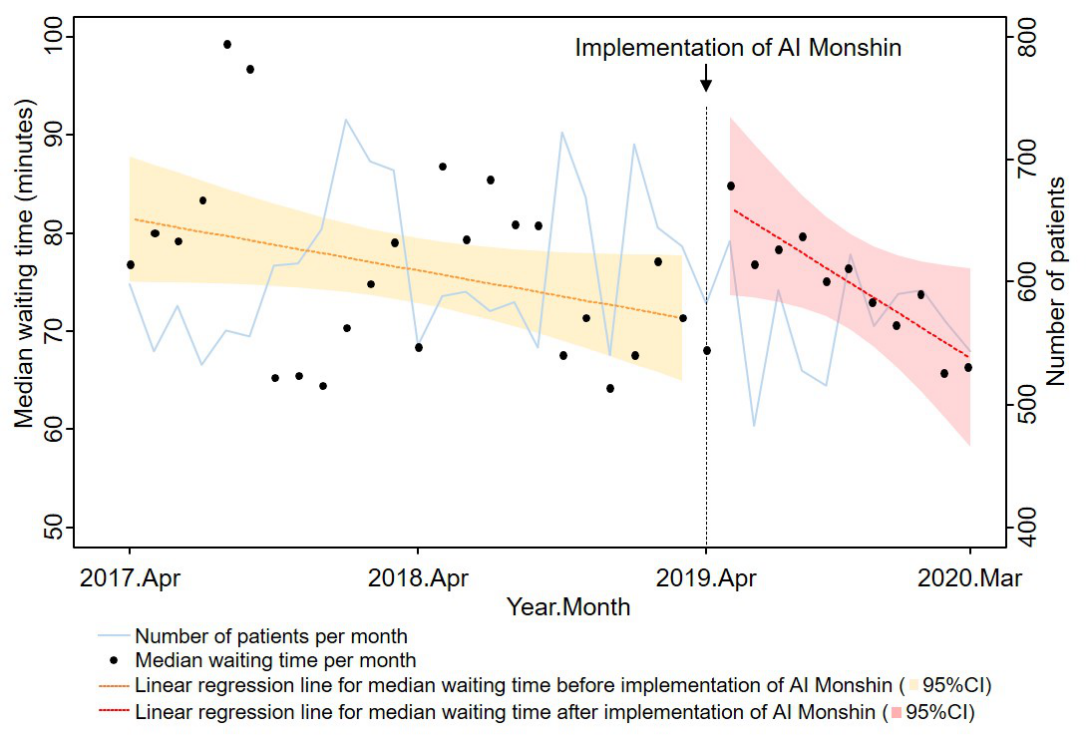
The trend in median waiting time and number of patients per month from April 2017 to April 2020.

Figure 2 shows the trends in the number of patients and median waiting time by month from April 2017 to April 2020. The drops in waiting time in February 2020 and March 2020 (the last two dots in Figure 2) could have been partially influenced by the efforts to mitigate the risk of the spread of coronavirus disease 2019 in the waiting room. In the interrupted time-series analysis, the underlying linear time trend was –0.4 minutes per month (*P*=.06, 95% CI –0.9 to 0.02), the level change at April 2019 was 40.6 minutes (*P*=.09, 95% CI –5.8 to 87.0), and the slope change starting in April 2019 was –1.1 minutes per month (*P*=.16, 95% CI –2.7 to 0.4).

### Supplemental Analysis

We added supplemental analyses for data from 9054 of 21,615 patient visits (41.9%) for whom the time of entry into the examination room was recorded in addition to the doctor's first recorded data entry. We calculated the assumed examination time as the time between patient entry into the examination room and the first recorded time of doctor’s data entry in the patient record, in 2491 of 6615 (37.7%) and 6563 of 15,000 (43.8%) patient visits before and after AI Monshin implementation (*P*<.001), respectively. The median assumed examination time after AI Monshin implementation (6.0 minutes, IQR 5.2) was significantly longer compared to the median assumed examination time before AI Monshin implementation (5.7 minutes, IQR 5.0; *P*=.003).

## Discussion

### Principal Results

To the best of our knowledge, this study is the first to evaluate the associated change from an AI-based automated medical history–taking system with patient waiting times at a general internal medicine outpatient department using an extensive data set of approximately 21,500 visits. Our results showed that the median waiting times before and after AI Monshin implementation were not significantly different from one another (*P*=.12). Moreover, the interrupted time-series analysis also showed no significant change in median waiting time (level change: 40.6 minutes, *P*=.09, 95% CI –5.8 to 87.0; slope change: –1.1 minutes per month, *P*=.16, 95% CI –2.7 to 0.4). In addition, we observed a slight increase in the examination time (including writing the patient record), with statistical significance (*P*=.003), after implementing AI Monshin.

### Limitations

This study had several limitations. First, there was the possibility of several confounding factors (such as staff skills, demographic changes, and case complexity) and other unmeasured confounding factors affecting the results. Therefore, we conducted time-series analysis in order to better interpret the results. Second, not all patients had data for examination start time. Thus, the waiting time in this study did not represent the actual waiting time in the waiting room. Moreover, the waiting time in this study may depend on when each doctor began entering data into the patient record; some doctors may prefer to enter data during patient examination, while others may prefer to enter data after the examination.

### Interpretation and Comparison With Prior Works

In our study, the use of AI Monshin did not reduce waiting time, contrary to our hypothesis for the usefulness of implementation of automated medical history taking. This negative result appears to be due to the amount of time automated medical history taking required and the characteristics of patients visiting general internal medicine outpatient departments without an appointment. As previously mentioned, automated medical history taking saves up to 15 minutes of overall patient time in the clinic when used at home in advance to the visit [21]. This may be because doctors were able to spare enough time to grasp the complete information taken by automated medical history taking and prepare for the examination; however, in this study, automated medical history taking was used in the waiting room right before examination. In this situation, the doctors may not have been able to make use of the large amount of data taken by automated medical history taking in just a few minutes. In addition, the completeness of automated medical history taking could be paradoxically associated to more examination time in specific situations. Consequently, the implementation of automated medical history taking actually led to longer examination times. According to a previous study [18], physicians estimated that the use of an automated medical history–taking device has the potential to become time consuming in low-complexity cases, in which the medical history is easily taken. In the setting of the small- to medium-sized hospitals in Japan, case complexity is usually low for patients visiting general internal medicine outpatient departments without an appointment [26,27]. We conducted this study in a single center (small- to medium-sized hospital) in Japan. Therefore, there may have been a selection bias since most of cases were assumed to be low-complexity cases, though no stratification of data into the degree of complexity was performed. Hence, the increase of examination time after AI Monshin implementation in this study is consistent with the assumption. This could explain why AI Monshin implementation failed to reduce patient waiting time in our study.

Although the waiting time was not reduced in this study, AI Monshin implementation may have optimized the quality of care. Previous reports [21] revealed that while some physicians used the same amount of time before and after the implementation of automated medical history taking, they could perform a more complete evaluation of the patient with automated medical history taking. We could not judge whether these quality changes occurred in this study because we did not survey changes such as the quantity and quality of patient-physician communication, patient satisfaction, or the accuracy of diagnosis. However, we can hypothesize that the implementation of automated medical history taking has the potential to optimize staff assignment. Indeed, after AI Monshin implementation, one out of the three nurses was replaced with a medical clerk, and thus an additional nurse was available to attend to patients. This shift in resources could have enhanced the quality of care. Moreover, because approximately half of first-visit patients revisit the outpatient department [27], the comprehensive patient history taken by AI Monshin may enhance the quality of care for subsequent visits. Moreover, using an AI-based automated medical history–taking system may improve the quality and quantity of data records, which otherwise vary among physicians [19], ultimately resulting in enhancement in the quality of medical care.

### Conclusions

The implementation of an AI-based automated medical history–taking system did not reduce the waiting time for patients visiting the general internal medicine outpatient department without an appointment. In addition, we noticed a slight increase in examination time after implementation. However, the implementation may have enhanced the quality of care by supporting the optimization of staff assignments. There may have been associations between case complexity and waiting time, examination time, and description time of patients. Therefore, we envision conducting further quantitative studies that take into account case complexity and that involve medical facilities of various sizes. Testing the effectiveness of automated medical history taking in reducing consultation time and explanation time between first versus second or subsequent visits is also a target issue for future study.

## Abbreviations

AI: artificial intelligence
